# The Value of Monitoring the Behavior of Circulating Tumor Cells at the End of Endocrine Therapy in Breast Cancer Patients

**DOI:** 10.3390/cancers10110407

**Published:** 2018-10-29

**Authors:** Katharina Pachmann, Stefan Schuster

**Affiliations:** 1 Medizinisches Labor Pachmann, SIMFO GmbH, Kurpromenade 2, 95448 Bayreuth, Germany; 2 SIMFO GmbH, Kurpromenade 2, 95448 Bayreuth, Germany; sschuster@simfo.de

**Keywords:** breast cancer, circulating tumor cells, endocrine therapy, longtime surveillance

## Abstract

After five years of endocrine therapy, patients with ER+ (estrogen receptor positive) breast cancer face the question of the benefit of further treatment. Ten years of endocrine therapy has been demonstrated to improve survival compared to five years. However, the individual benefit of continuation remains unclear. Therefore, markers for predicting benefit from endocrine treatment and extended endocrine treatment are desperately needed. In this study the dynamics over time of the tumor cells circulating in peripheral blood of patients, circulating tumor cells/ circulating epithelial tumor cells (CTC/CETC), as the systemic part of the tumor were investigated in 36 patients with ER+ primary breast cancer. CTC/CETCs were monitored serially during and after endocrine therapy. After termination of endocrine therapy 12 patients showed an increase in CTC/CETCs, with 8 of 12 suffering relapse. No change or a reduction was observed in 24 patients, with 2 of 24 suffering relapse. Initial tumor size was marginally prognostic (*p* = 0.053) but not nodal status nor the mere number of CTC/CETCs. Only the trajectory of CTC/CETCs was a statistically significant predictor of relapse free survival (increasing cell numbers: mean = 940 days vs. stable/decreasing cell numbers mean not reached). Individual cases demonstrated that an increase of CTC/CETCs after discontinuation of tamoxifen therapy could be stopped by resuming the endocrine therapy.

## 1. Introduction

Until recently adjuvant chemotherapy was given to almost all primary breast cancer patients in order to prevent metastasis formation [1]. Since most chemotherapeutic agents are mainly active against proliferating cells it has long been known that this leads to overtreatment, especially in patients that have estrogen receptor positive breast cancer with a low fraction of proliferating cells [2]. Therefore, expression analyses from the primary tumor comprising genes which are involved in multiple cellular and cancer-associated processes such as cell cycle, proliferation, invasion, angiogenesis, and metastasis formation [3,4] were developed and the term precision oncology was used to describe the selection or deselection of adjuvant chemotherapy guided by these expression profiling tools. However, there is still no consensus as to whether these new tools will definitely be helpful. According to the German Institute for Quality and Efficiency in Healthcare (IQWiG) [5] to date there is no evidence neither of benefit nor harm of a biomarker-based strategy for the decision for or against chemotherapy in primary early stage estrogen receptor positive breast cancer. Therefore, it remains the decision of the patient who may mull for years over whether she has made the right decision.

More recently mutation analyses used to guide targeted therapy in different cancer types are referred to as precision medicines but, so far, relevant mutations are difficult to detect in early breast cancer and as a matter of principle the success of such an approach has recently been challenged [6].

In contrast, endocrine therapy was established as the earliest and most successful targeted (precision) therapy in hormone receptor positive breast cancer and it seems to have the strongest impact on relapse-free and overall survival [7].

Endocrine therapy is unequivocally accepted in all hormone receptor positive patients in contrast to chemotherapy [2] and currently is scheduled for five years. Obviously, treatment with Tamoxifen benefits these patients [8]. But even after five years of effective therapy with selective estrogen receptor modulators (SERM), relapses still occur. The ATLAS (Adjuvant Tamoxifen: Longer Against Shorter) study demonstrated that 10 years administration of Tamoxifen is superior to that of 5 years [9] which has been confirmed recently by the aTTom study [10].

Some of the side effects of tamoxifen treatment like thrombotic events and embolism as well as endometriosis and endometrial cancer are serious [11], and for most women five years of treatment with hormone blockers already poses a problem which often requires motivating discussions with the patient. For the individual woman it remains unclear whether she may benefit from extended endocrine therapy and patients and care providers bemoan that during follow-up neither frequent serum tumor marker determination nor imaging for early detection of relapse is recommended [12], with only local monitoring being advised. Serial follow up of the number of the circulating cells suspect to be of tumor origin (CETC/CTC) in blood during and after completion of chemotherapy [13] may contribute to closing this diagnostic gap.

It is well known that cells are able to break away from malignant tumors and circulate in blood. These macroscopically undetectable circulating tumor cells remaining in the body can contribute to (the development of) metastases [14]. Our results indicate that tamoxifen may not kill most cancer cells but most probably drives them into dormancy [15]. They may then become active after the termination of therapy, leading to tumor growth locally and/or as distant metastases.

**Figure 1:**
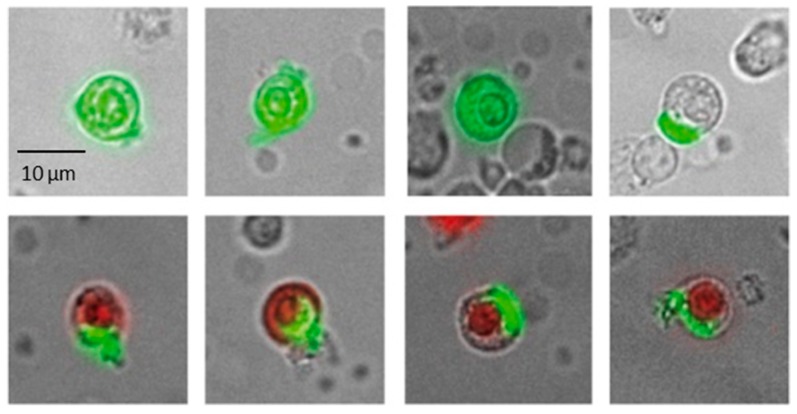
Typical gallery of images of live (EpCAM fluorescein-isothiocyanate (FITC) green) and dead (green and red Propidium Iodide Uptake) tumor suspect cells of one patient. Dead cells are not included into the cell count.

Using the maintrac^®^ approach, carcinoma-derived cells can be detected without enrichment among the other blood cells due to the surface expression of EpCAM (epithelial cell adhesion molecule) specific for epithelial cells (Figure 1) [16].

Although benign epithelial cells can enter the bloodstream through various events including injuries, burns, inflammatory diseases, and surgical procedures [17,18], these cells are usually rapidly removed from the circulation, whereas epithelial tumor cells can survive up to 20 years [19,20] and can recirculate in blood [21]. These cells can be monitored in peripheral blood not only during chemotherapy [14] but it is also possible to follow their evolution during endocrine treatment [22]. An increase in cell numbers generally indicates increased tumor activity and is associated with a poor prognosis, whereas a decrease in cell numbers indicates successful treatment and a good prognosis [23]. Thus, the key part in monitoring therapy with the maintrac^®^ method is not a one-off measurement, but the analysis of the dynamics of cell numbers over a prolonged period.

The present report demonstrates that this is also true after discontinuation of endocrine treatment. Most importantly, we show that an increase in cell numbers after the end of endocrine treatment is of highly significant clinical relevance but that a (re-)uptake of therapy can lead to re-decreasing CETC and to potentially minimize the risk of developing metastases. Thus, it is possible in early breast cancer patients to identify patients for which a prolonged endocrine therapy is necessary within a period of weeks after terminating the previous round of medication.

## 2. Results

The patient population comprised 36 patients with hormone receptor positive tumors who received tamoxifen or an aromatase inhibitor as endocrine therapy for hormone receptor positive primary breast cancer for up to 5 years. The median time of initial diagnosis was 7 years prior to assessment. The median observation period after completion of endocrine therapy was 608 days (range, 14–2, 159 days). Further patient characteristics are listed in Table 1.

20 patients had T1 (55%) tumors and 15 patients T2 and larger (42%), 1 with unknown size (3%). Lymph node status was N0 in 24 patients and N1–2 in 12 patients.

**Figure 2:**
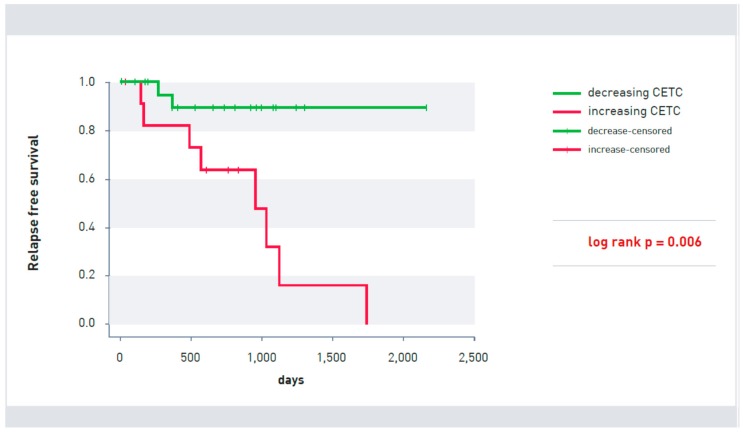
Kaplan-Meier survival curve of patients with increasing (red line) and decreasing (green line) CETCs (*p* = 0.006). Patients without signs of relapse at the last visit were censored.

CETC behavior in 36 patients was significantly correlated with recurrence after endocrine therapy (*p* = 0.006). After the end of endocrine therapy, an increase in CETCs was observed in 12 patients, of whom 8 patients suffered relapse. The cell count remained stable or decreased in 24 patients even after completion of endocrine therapy. In this group, two patients relapsed during the course of the study (Figure 2).

**Figure 3:**
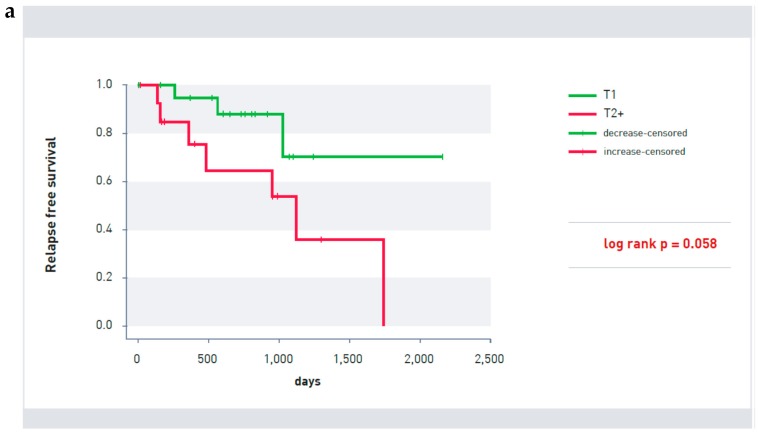

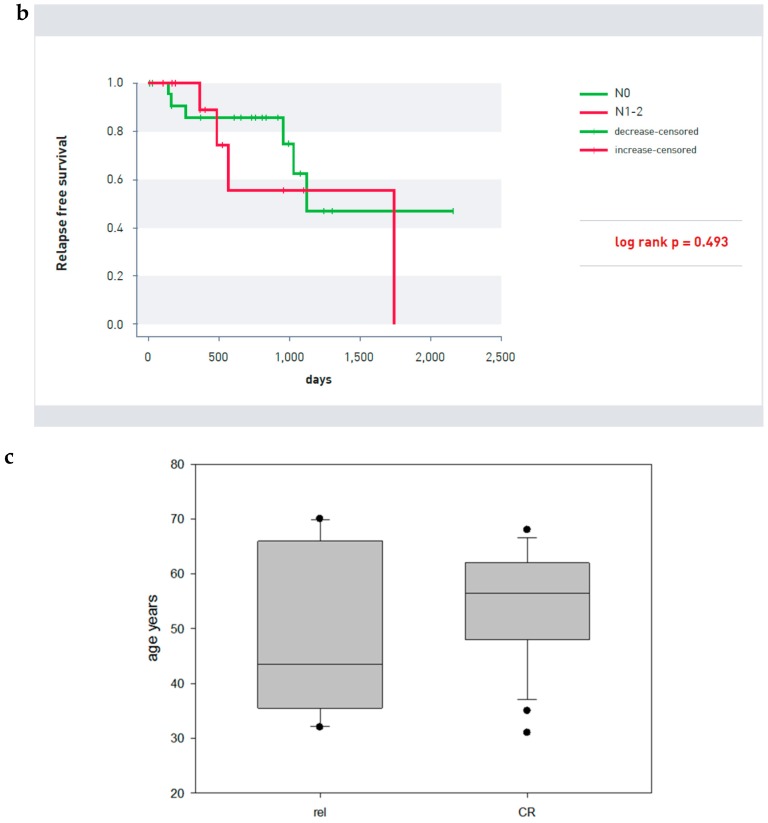
(**a**) Kaplan-Meier survival curve of patients with T2 as well as larger (red line) T1 size tumors (green line) (*p* = 0.058). (**b**) Kaplan-Meier survival curve of patients with (red line) and without lymph node involvement (green line) (*p* = 0.493). (**c**) Age distribution of patients with (R) and without relapse (CR) (Mann-Whitney U test *p* = 0.512).

Established prognostic factors such as tumor size or nodal status were not predictive of prognosis nor were age in this patient population after the end of endocrine therapy. While tumor size indicated a tendency (*p* = 0.058; Figure 3a) for tumors bigger than T1 size to have a poorer prognosis (only one patient had a tumor bigger than T2), neither the nodal status (Figure 3b) nor age (Figure 3c) was significant for recurrence-free survival after endocrine therapy in this small patient cohort.

**Figure 4:**
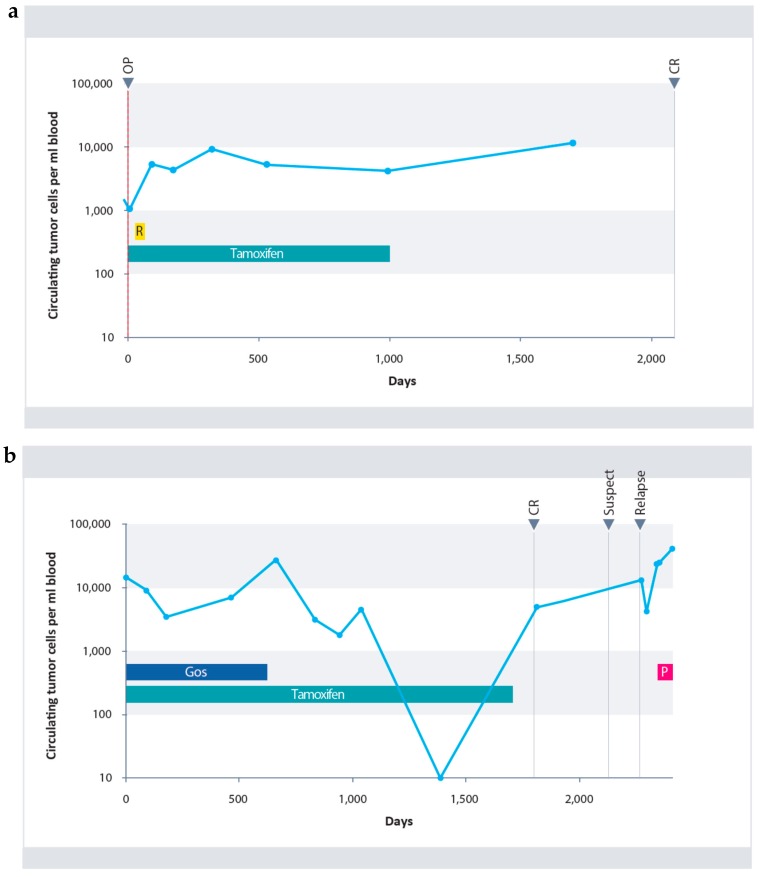
(**a**) Course of CETCs in a patient with invasive ductal carcinoma (pT1bpN2aM0). Initially, an increase in cell numbers under radiotherapy was observed (hatched in yellow). Cell numbers remained primarily constant under further Tamoxifen therapy (blue). Follow-up samples of the patient revealed macroscopic complete remission (CR). (**b**) Course of CETCs in a patient with invasive lobular carcinoma who was treated with neoadjuvant therapy (ypT1a(m)pN1aM0). Under combined therapy comprising of Goserelin (hatched in dark blue) and Tamoxifen (blue), an initial decrease in CETCs was observed, followed by a renewed increase during further combination therapy. Under monotherapy with Tamoxifen a continuous decrease in CETCs was observed followed by a renewed increase of CETCs after completion of endocrine therapy. At that time the patient was macroscopically still in complete remission. After additional 10 months recurrence was confirmed and after mastectomy was treated with Paclitaxel (purple).

Typical courses of circulating tumor cells of two patients from the study population are shown in Figure 4a,b.

The first patient showed an increase in CETCs during radiotherapy. Subsequently, her number of CETCs remained fairly constant for three years under Tamoxifen treatment and even 2 years after terminating treatment. She is relapse free as of the present day (Figure 4a). The second patient demonstrated considerable variation of CETCs under the 5-years-therapy with Tamoxifen (Figure 4b). Due to pre-menopausal status the patient received the GnRH analogue Gosereline in addition to Tamoxifen, for the first 2 years. Under further Tamoxifen therapy complete elimination of CETCs from the circulation was observed.

However, four months after completion of endocrine therapy, the patient’s cell count increased drastically, although there was no indication of relapse at examination. The numbers continued to increase and 10 months later, relapse was detected and after mastectomy, was treated with weekly Paclitaxel.

Additionally, 3 cases are presented where Tamoxifen therapy was terminated due to side effects but subsequently resumed upon recommendation.

**Figure 5:**
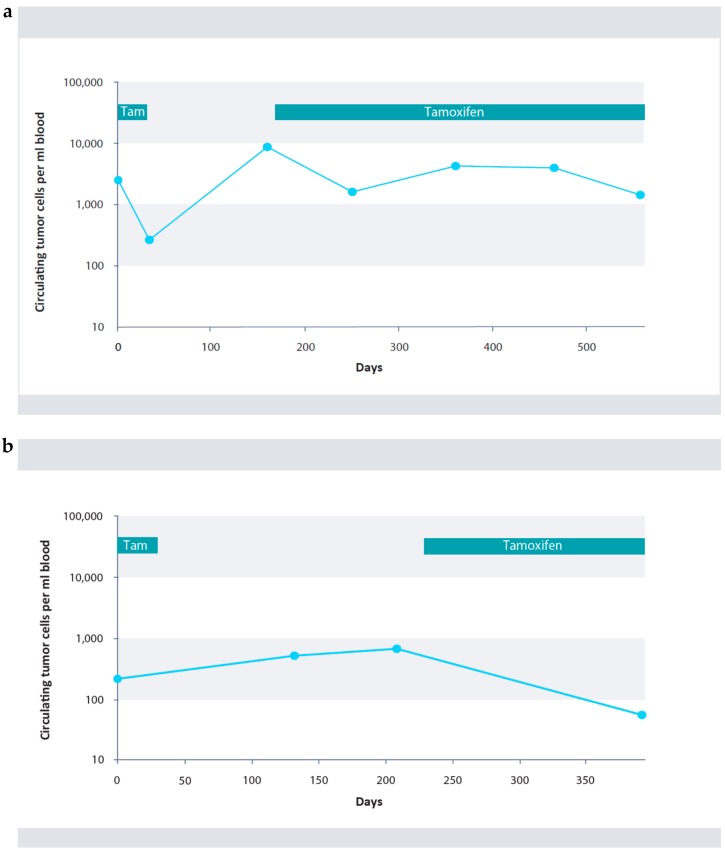

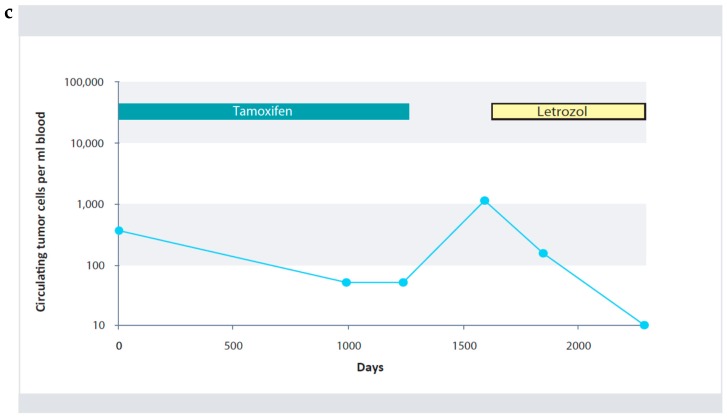
(**a**) Breast cancer (pT1cN0M0G2, HR+, Her2/neu: neg). 4 times TC (taxane and cyclophosphamide). Tamoxifen interrupted because of side effects. Reiinitiation of Tamoxifen. (**b**) Breast Cancer (pT2pN0M0). 6 times FEC (fluoruracil, epirubicine and cyclophophasmide, Herceptin, Radiatio, Tamoxifen interrupted. Reiinitiation of Tamoxifen. (**c**) Breast Cancer (pT2pN2apM0G3) Er+ PR+ Her2/neu FISH neg, 3 times FEC, 3 times Docetaxel, Radiatio, Tamoxifen interrupted, then Letrozol.

In the three patients shown in Figure 5a–c we were able to show that if endocrine therapy was reinitiated (Tamoxifen or aromatase inhibitor, such as Letrozol) after an increase of CETC during adjournment of therapy, cell numbers re-declined and the patients have remained relapse free so far.

## 3. Discussion

Detection and serial monitoring of cells expressing the epithelial surface molecule EpCAM in peripheral blood of patients with hormone receptor positive breast cancer was used to follow the fate of these cells during endocrine e treatment. Although expressed by most cancer cells, EpCAM is not confined to malignant cells but also found on normal epithelial cells. Dead cells or macrophages may take up anti-EpCAM antibody non-specifically but this was excluded by analyzing only live cells with strictly surface located expression. If normal epithelial cells are accidentally released into blood they are not expected to increase in numbers. In contrast, epithelial tumor cells are assumed to be shed into blood continuously during tumor growth [24].

Therefore, a single determination of the number of epithelial cells suspect to be of tumor origin, circulating in blood which we have termed CETCs, may have little prognostic relevance in primary tumors. However, repeated blood drawing allows for the detection of CETCs at any time point during the course of disease making serial monitoring possible. An increase in numbers of these cells generally has been shown to indicate increased tumor activity and was associated with poor prognosis, whereas a decrease in cell numbers indicates successful treatment and a good prognosis as shown in various studies under different therapies involving more than 650 patients [17,18,22,25,26,27].

The present results where a relapse after the end of endocrine therapy was observed in 8 of 12 patients with increasing cell numbers and only in 2 of 24 patients with decreasing or stable cell numbers confirm the above observations also apply in the situation of endocrine maintenance therapy.

This allows to individually monitor patients, particularly if a decision is pending on whether to terminate e.g., endocrine therapy. Careful and consequent examination of patients to determine whether cell numbers have a tendency to increase again after the end of therapy, could reveal increased tumor activity at an early stage. Quantitative serial monitoring of living epithelial tumor cells can then help to assess whether extended endocrine therapy in these patients has an impact on their circulating cells and the course of disease. The observation of the nodal status not being of significance was initially surprising; however, this finding may have been related to the long observation period. After initial diagnosis, the median observation period of patients was 7 years. It is known that in patients with a high nodal status relapse occurs at an early stage [28]. In the present study, this patient cohort was underrepresented. This most probably also applies for tumor size, although a tendency for relapse after endocrine therapy was observed for larger tumors.

The measurement of CETCs in blood provides breast cancer patients the opportunity to monitor their course of the disease. After the completion of endocrine therapy, the observation of increasing CETCs in a routine setting could lead to two plausible scenarios: Either the increase should lead to further diagnostic measures allowing for the early initiation of interventional treatment or the direct reuptake of the endocrine treatment. The first scenario has the advantage that therapeutic interventions can be taken within the established guideline if a macroscopic relapse is detected. The second allows for detecting tumor activity using CETCs already when even highly sensitive imaging is not able to detect a relapse but it is not included within the guidelines. Both scenarios offer the possibility that a relapse will be detected earlier. To determine if this leads to longer survival of the patients, further studies are needed.

## 4. Methods and Patients

The patients were recruited at the University Hospital of Jena between 2001 and 2012. All patients had estrogen receptor positive breast cancer, confirmed by histopathology. Only patients without metastasis were included in this study, independent from their menopausal status. Patients received either adjuvant or neoadjuvant treatment. This population represents a subgroup of patients that have been monitored during endocrine treatment and which has been published previously [22].

Ethical approval was given by the University of Jena on 13 September 2002, ethical Code 0921-08/02. The maintrac^®^ approach was used for detection and quantification of CETCs, as reported previously [23]. CETCs were determined from whole blood without enrichment. Blood samples were drawn into blood count tubes with ethylene diamine-tetra-acetic-acid (EDTA) for enumeration of CETCs. In brief, 1 mL blood was subjected to red blood cell lysis using 15 mL of erythrocyte lysis solution (Qiagen, Hilden, Germany). The solution was then cooled for 15 min, spun down at 700 g and re-diluted in 500 mL of PBS-EDTA. 5 µL of fluorescein-isothiocyanate (FITC)-conjugated mouse anti-human epithelial antibody (EpCAM) (Miltenyi Biotec GmbH, Bergisch Gladbach, Germany) was added and incubated for 15 min at 4 °C. Subsequently, the samples were diluted with 430 µL PBS-EDTA and then stored over night at 4 °C. A defined volume of the cell suspension and propidium iodide (Sigma-Aldrich, St. Louis, MO, USA) was transferred to a 96-wells plate (Greiner Bio-one, Kremsmünster, Austria), evaluated with a fluorescence scanning microscope scanR (OLYMPUS EUROPA SE & CO. KG, Hamburg, Germany,) and visually examined for the presence of fluorescence and cell morphology. Only live CETCs were counted with positive EpCAM fluorescence, lacking nuclear PI staining, and with intact morphology (Figure 1). Two types of quality controls were used for ensuring the consistent analysis of samples: Isotype control, as a negative control to measure the level of non-specific background signal and fluorospheres (Flow-Check 770, Beckman Coulter, Brea, CA, USA) for the daily verification of optical components and detectors. The number of CETCs in blood was repeatedly measured during and after completion of endocrine therapy. An increase or decrease in cell numbers was assumed as soon as cell numbers were altered by at least a factor of five following the termination of endocrine treatment. The mean period for CETC to increase was 631 days (range 60 to 1401 days).

Statistical analyses were calculated by the Kaplan-Meier method and differences of relapse free survival were compared by the log-rank test using SigmaPlot 13 for Windows (Systat Software Inc., San Jose, CA, USA). *p* < 0.05 was considered to indicate a statistically significant difference.

## 5. Conclusions

In recent years, Tamoxifen therapy has clearly been demonstrated to be beneficial for patients with hormone receptor positive breast cancer despite known side effects, such as thrombosis, postmenopausal symptoms, and an increased long-term risk of endometrial cancer. Aromatase inhibitors have become an option in the treatment of hormone receptor positive breast cancer, especially if the side effects of Tamoxifen treatment are no longer tolerable.

CETC may provide valuable insights in the tumor activity to track relapses early and also to distinguish between effective and less effective therapies. Having observed fluctuations in cell numbers in patients with an irregular Tamoxifen intake, we found that in patients with increasing cell numbers after stopping endocrine treatment re-intake of Tamoxifen or switch to aromatase inhibitors again reduced the number of circulating tumor cells and no patient suffered from relapse in the observation period. This led us to assume that a re-uptake of endocrine therapy may be beneficial in patients with increasing cell numbers and might be able to prevent or at least delay recurrence. These first results are already promising.

Further studies are needed to clarify if for patients with increasing cell numbers a resumption of endocrine therapy is advantageous to prevent metastasis formation. Currently an interventional trial is planned to investigate this option based on the results of CETCs using the maintrac^®^ approach. This would then provide a basis by which patients and therapists can make an individual decision about whether endocrine therapy should be continued or reinitiated. The response to treatment in each individual patient may be more important than the initial clinical or biologic features because these characteristics can change during the course of the disease.

## Figures and Tables

**Table 1:** Patient population.

**Patient Population**		
Number of patients Age	36 31–80	
Relapse	10	
Mean age patients relapsed	43	Ns
Mean age patients in complete remission	56	
Observation period after endocrine therapy	608 days *	
Observation period after initial diagnosis	2281 days *	
**CETCs**		
Increasing	12	Rel 8
Decreasing/Constant	24	Rel 2
**Tumor Size**		
T1	20	Rel 3
>T2	15	Rel 7
Unknown	1	
**Nodal Status**		
N0	24	Rel 6
N1–2	12	Rel 4

* Observation periods are reported as median values. CETCs = circulating epithelial tumor cells. (Ns = not significant, Rel = Relapse).
